# The Significance of Cooperation in Interdisciplinary Health Care Teams as Perceived by Polish Medical Students

**DOI:** 10.3390/ijerph20020954

**Published:** 2023-01-05

**Authors:** Aleksandra Bendowska, Ewa Baum

**Affiliations:** 1 Department of Social Sciences and the Humanities, Poznan University of Medical Sciences, 60-806 Poznan, Poland; 2 Division of Philosophy of Medicine and Bioethics, Poznan University of Medical Sciences, 60-806 Poznan, Poland

**Keywords:** interdisciplinary team, multidisciplinary team, transdisciplinary team, healthcare team, interdisciplinary medical education

## Abstract

Teamwork, as the preferred method of cooperation in healthcare, became prevalent in the 1960s, and since then has been universally recognized as a measure to improve the quality of healthcare. Research indicates that medical care based on interdisciplinary cooperation is associated with increased patient safety, lower hospitalization rates, and reduced rates of complications and medical errors. Furthermore, it enhances the coordination of care and improves patient access to medical services. This model of providing medical care also results in considerable benefits for medical professionals. These include greater job satisfaction and a reduced risk of professional burnout syndrome. Aim: The aim of the study was to explore the opinions of medical students with regard to cooperation in the interdisciplinary team, as well as the factors affecting the formation of opinions. Material and methods: The study was conducted using the Polish version of the questionnaire Attitudes Towards Interprofessional Health Care Teams. The study involved 1266 participants, including students of medicine (*n* = 308), midwifery (*n* = 348), nursing (*n* = 316) and physiotherapy (*n* = 294). Results: According to the opinions of the students participating in the study, the therapeutic process based on the interdisciplinary model improves the quality of medical care provided, increases patient safety, and improves communication between members of the therapeutic team. The factors affecting the assessment of cooperation in interdisciplinary medical care teams included the faculty and the year of studies, gender, as well as participation in the multidisciplinary courses. Conclusions: Students recognize the need for interdisciplinary medical teams. The training of future medical professionals should incorporate the elements of interprofessional education. This form of education allows students to develop both a professional identity and identification with their own profession, as well as encourages teamwork skills and shapes the attitude of openness towards representatives of other medical professions. However, in order to provide the students with the relevant knowledge, skills and competencies, it is essential to respect their diversity in terms of the faculty, as well as to account for the impact of gender and the year of studies which may affect their readiness to engage in teamwork.

## 1. Introduction

The World Health Organization (WHO) defines a team as a group of two or more individuals who interact dynamically, independently and adaptively towards a common and valued goal or mission, and who have been assigned specific roles or functions to perform at a specified time [1].

In healthcare, teams are frequently formed to address challenging clinical issues by means of introducing innovative solutions. The underlying rationale is the fact that the decisions and actions undertaken by the team should more effectively resolve multidimensional issues [2].

Depending on the degree of responsibility for patient care and the level of interaction among team members, in healthcare, a distinction is made between the interdisciplinary team (IDT), multidisciplinary team (MDT) and transdisciplinary team (TDT) [3]. These terms are often used interchangeably, although they differ both etymologically and semantically.

A multidisciplinary team is usually perceived as a team in which each professional operates within their specific scope of expertise and interacts formally. In turn, interdisciplinary teams are characterized by a greater overlap of professional roles, formal and informal communication and joint problem solving for the benefit of the patient. Furthermore, a transdisciplinary team involves even more overlapping of roles, for instance, where one team member may assume the role of a team leader responsible for coordinating the entire patient care.

Multidisciplinary teams may evolve into interdisciplinary teams. Although frequently used interchangeably, these two terms denote markedly different modes of coordination, cooperation and communication. In the multidisciplinary model, each discipline sets patient care goals according to its field of expertise, independent of other professionals. In contrast, professionals cooperating in interdisciplinary teams rely on expert opinion from each discipline, which results in the creation of common patient goals [4,5].

Improving the healthcare system requires simultaneous pursuit of four goals: enhancing the quality of care provided, improving the population’s health status, reducing healthcare costs and increasing job satisfaction among healthcare professionals [6]. Numerous studies indicate that the above-mentioned goals may be achieved by means of cooperation in interdisciplinary teams.

As pointed out by Littlechild and Smith [7], cooperation in interdisciplinary teams increases productivity, contributes to the better use of team members’ skills and leads to a greater feeling of individual responsibility for achieving the objective, as well as ensures holistic patient care, stimulates creativity and results in innovative solutions in patient care.

In 2010, the WHO in their report stated that interdisciplinary teams were associated with better outcomes in the areas of family medicine, treatment and control of infectious disease, as well as humanitarian relief. Furthermore, such teams were found to be more effective in terms of controlling epidemics and non-communicable diseases ([8], p. 14). Facilities where health professionals cooperated reported reduced rates of medical complications and errors, decreased mortality rates, shorter hospitalization periods for patients, and medical professionals reported fewer communication misunderstandings ([8], p. 18). In addition, subsequent research demonstrated improvements in patient access to medical care, proper coordination of care, and increased patient safety [9].

According to the studies, the introduction of cooperation based on interdisciplinary teams reduces the incidence of complications in the departments of internal diseases [10]. Moreover, a decrease in complication rates due to intravenous cannula insertions was also reported, which was attributed to the participation of an infectious diseases specialist and an epidemiological nurse who were present in the therapeutic team [11]. Additionally, a reduction in the incidence of adverse drug reactions was also reported. This, in turn, was associated with the presence of a pharmacist in the team [12] as well as with the implementation of interdisciplinary ward rounds [13].

The interdisciplinary team represents one of the basic forms of patient care in palliative and hospice care [14]. In fact, the holistic approach to both palliative and hospice care constitutes an essential element of patient care. The holistic approach contributes to better symptom control, reduced strain and improved quality of life for patients’ carers, as well as increased efficiency in achieving care goals. Additionally, such an approach facilitates going through the process of dying in the environment selected by the patient [15]. Furthermore, patients’ carers tend to assess the care better with regard to effectiveness in pain management and communication skills of the healthcare professionals, as well as in terms of care and respect for dignity [16]. Such an attitude has also been associated with enhanced motivation, greater job satisfaction and a perspective of continuous development among medical professionals [17].

As emphasized by Modlińska [14], the experience of working in an interdisciplinary team allows to perceive the patient as one entity—the “whole” person, rather than individual body parts, or affected systems. The cooperation of a team consisting of a physician, nurse, healthcare provider, chaplain, social worker and family members allows for more effective fulfillment of the patient’s needs and contributes to better quality of their lives. Additionally, it facilitates the identification of areas in the care system that require improvement, which is not always possible with the traditional approach, where the focus is on a specific, limited area.

Due to the process of aging affecting the Polish population, a demand for comprehensive medical care has emerged. In this respect, the geriatric approach, comprising the interdisciplinary cooperation of a physician, nurse, physiotherapist, psychologist, medical carer, community therapist, social worker and, if required, also a speech therapist, dietician, pharmacist or chaplain has been positively evaluated. The geriatric team approach involves a comprehensive assessment of the health problems, physical fitness, and mental condition, the level of social support, including family support, economic condition and living conditions of the geriatric patient. Moreover, the aforementioned measures implemented by the interdisciplinary geriatric team are aimed at improving the level and quality of health services and improving the functional state and quality of life of the patient [18,19].

Apart from geriatric, hospice and palliative care, interdisciplinary teams are recommended as a form of medical care in pediatrics [20], in patients suffering from infantile cerebral palsy [21], in the course of obesity [22] and diabetes mellitus [23], as well as in gynecological conditions, such as infertility [24], premenstrual syndrome and premenstrual dystrophic disorders [25] and in the prevention of pressure ulcers [26].

The changing population structure constitutes a challenge not only for the healthcare system, but for the entire education system of future medical professionals. According to the analysis conducted by the Organisation for Economic Co-operation and Development (OECD), the ability to formulate clear objectives for cooperation in interdisciplinary teams, find new opportunities and identify solutions for complex problems will become indispensable in the years to come. Furthermore, education should focus not only on preparing students for their future profession, but it must also provide them with the necessary skills to become active, responsible and engaged citizens [27]. The report also emphasizes the fact that it is essential for pupils and students to develop the ability to think and act in a systemic way, which is based on recognizing the interconnections and relationships between ideas, views and positions. According to the authors, it may be achieved by means of cooperation, in which participants exchange their knowledge in order to generate new ideas. This assumption is fulfilled by interprofessional education, which occurs when students/representatives of at least two different disciplines/fields study together about the scope of their competencies and skills, from and with one another, with the objective of productive cooperation and improving the quality of patient care ([8], p. 15).

The recommendations included in The Future of Education and Skills. Education 2030. The Future We Want are in line with the 2010 WHO document entitled Framework for Action on Interprofessional Education & Collaborative Practice. The guidelines outlined the condition of interdisciplinary collaboration worldwide, identified the mechanisms which shape successful team cooperation and provided a range of actions for policy makers to apply in their local healthcare system. The aim of the report was to provide strategies and ideas to help national health policy makers implement elements of interprofessional education and cooperation in interdisciplinary teams ([8], p. 20).

The authors emphasize that interprofessional education and establishing the healthcare system on the principles of interdisciplinary collaboration do not constitute the solution to every challenge healthcare may face. However, if applied appropriately, they have the potential of providing the staff with the skills and knowledge required in order to address the challenges of the increasingly complex healthcare system. Therefore, our aim in this study was to explore the opinions of medical students and future medical professionals, with regard to cooperation in the interdisciplinary team, as well as the factors affecting the formation of opinions. We believe that the knowledge of students’ approach to interdisciplinarity will allow for more effective adaptation of educational programs and didactic methods necessary to acquire the skills of cooperation between representatives of various medical professions.

## 2. Materials and Methods

The study involved the diagnostic survey method, using the survey technique in the form of a questionnaire. The research tool consisted of a Polish version of the questionnaire Attitudes Towards Interprofessional Health Care Teams (ATHCT) [28]. A note was added at the end of the questionnaire which referred to the gender, age, year of studies and faculty, as well as to the place of residence.

In order to verify the research tool, to test the intelligibility and clarity of the questions, a pilot study was conducted prior to the main research, which involved a group of 70 individuals. In addition, the questionnaire was subjected to validation, consisting of two parts: the translation and the assessment of the accuracy of the newly translated instrument. The original version of the research tool was translated into Polish by two independent English interpreters. In order to determine the reliability of the questionnaire obtained following the translation into Polish, the Cronbach’s alpha coefficient was determined, which was above 0.6. Following this test, the final version of the questionnaire was established, using which the actual study was conducted.

The employed version of the ATHCT questionnaire, comprised 14 statements with regard to the impact of cooperation in the interdisciplinary team on the quality of medical care provided, as well as the costs associated with this form of diagnostic and therapeutic management. The questionnaire involved a 5-point Likert scale, where the following scores were used: 1—strongly disagree; 2—disagree; 3—neutral; 4—agree; 5—strongly agree. It was accepted that the higher the mean score obtained in the questionnaire, the more significant the importance of cooperation in interdisciplinary teams.

The students completed the questionnaire independently and anonymously, and the note was included at the end of the form. Due to the prevailing SARS-CoV-2 pandemic, the survey continued using Google Forms software as of March 2020. It incorporated the electronic version of the questionnaire identical to the paper version. The traditional, paper version of the survey was submitted by 545 respondents. A total of 721 students participated in the survey using the electronic version.

Before starting the research, The Bioethics Committee of the Poznań University of Medical Sciences issued an opinion that there was no medical experiment and allowed the project to be carried out.

### Statistical Methods

The assumption of homogeneity of variance was tested by means of Levene’s test. Comparisons of arithmetic means between groups were performed using one-way analysis of variance, or Welch’s F test (if assumptions were not met). For multiple comparisons (post-hoc), Tukey’s HSD test in the version for unequal numbers was used.

The study applied parametric tests referring to the methodology used by the author of the original ATHCT survey questionnaire [28]. The results were considered statistically significant when *p*-value < 0.05.

The analysis was performed using Statistica v.10.0 software from StatSoft Polska (Krakow, Poland).

## 3. Results

The survey was conducted between March 2019 and September 2020 with 1266 students from Poznan University of Medical Sciences (Poland). It involved students from the faculties of medicine, midwifery, nursing and physiotherapy. In order to evaluate the impact of the academic training on the students’ attitudes with regard to interdisciplinary medical teams, both students who have just started their university education, as well as final-year students, were included in the study. The former group participated in the research during the first months of the academic year (September–December), whereas the final-year students completed the survey between March and June.

The study comprised representatives of only four faculties—nursing, midwifery, physiotherapy and medicine. We excluded students of other medical faculties, e.g., pharmacy, occupational therapy, electroradiology due to the fact that in Polish healthcare system, the professionals who most frequently comprise the therapeutic team include nurses, midwives, physicians, and physiotherapists (Table 1).

Midwifery students constituted 28% of the studied population, nursing students—25%, medical students—24%, and physiotherapy students—23%.

Women were predominant among the study population (more than 81%), persons between 18 and 20 years of age represented 57% of the analyzed group. The mean age was 20.8 ± 2.2 years. The youngest participant was a student aged 18, and the oldest participant was 33 years old.

The place of residence constituted a differentiating factor in the research. The majority of subjects lived in the countryside (*n* = 364). Another group included residents of large cities (more than 500,000 inhabitants; *n* = 335), cities with a population under 50,000 (*n* = 296) and cities with a population between 50,000 and 500,000 (*n* = 271).

In the course of their studies at Poznan University of Medical Sciences, only 34% of the study participants attended classes with medical students from other faculties. Most frequently, such courses were the standard curriculum classes or elective courses. Nevertheless, the students were also allowed to fill in their own responses in the survey, therefore, among the classes involving students from other faculties which had not been included in the questionnaire, the participants listed meetings of the scientific associations, students’ scientific societies, student self-government, university choir or sport activities.

It is worth noting that only 44% of the students participating in the above-mentioned classes stated that the teachers used methods aimed at encouraging interaction and cooperation in teams consisting of students from different medical faculties. Moreover, 37% of students did not observe such a form of encouragement on the part of the teacher, and nearly one in five (19%) had no opinion. Most frequently, students indicated that such courses lasted 1–15 h.

### 3.1. The Perception of the Significance of Cooperation in Interdisciplinary Health Care Teams according to the Faculty

According to the scores provided by the students, cooperation in interdisciplinary healthcare teams was perceived as significant (3.95 ± 0.41). Additionally, it was found that the faculty affected (*p* < 0.001) the differences in the perception of collaboration in interdisciplinary healthcare teams.

Providing medical care by the interdisciplinary team was found to be most beneficial according to the nursing students, in turn, it was not as significant for physiotherapy students, midwifery students and medical students. Nevertheless, the differences observed in the opinions of the students were minor (Table 2).

The analysis of multiple comparisons revealed that the faculty affected the differences in the perception of the significance of cooperation in interdisciplinary teams only among the nursing and medical students (*p* < 0.001), as well as among the nursing and midwifery students (*p* < 0.001). No significant differences with respect to the discussed issue were observed between the opinions of the students from other faculties.

### 3.2. The Perception of the Significance of Cooperation in Interdisciplinary Medical Teams according to the Year of Studies

Students in their final year of studies—6th year of the long-cycle program in medicine, 3rd year of undergraduate programs in midwifery, nursing and physiotherapy—perceived providing medical care by the interdisciplinary medical team as more significant in comparison to students who had just started their university medical education (Table 3).

### 3.3. The Perception of the Significance of Cooperation in Interdisciplinary Medical Teams according to the Participation in Multidisciplinary Courses

The students who participated in courses involving representatives of other medical faculties (multidisciplinary education) perceived cooperation in interdisciplinary teams as slightly more significant as compared to students who did not participate in such classes (Table 4).

It should be noted that in this group, the highest mean scores (4.09 ± 0.43) were obtained by the students whose lecturers encouraged interaction and cooperation in groups involving students from different faculties during classes, which constitutes a characteristic element of interprofessional education.

### 3.4. The Perception of the Significance of Cooperation in Interdisciplinary Health Care Teams according to Gender

The analysis revealed statistically significant differences between the perception of cooperation in interdisciplinary medical teams and the gender of the participants. In fact, female respondents perceived such collaboration as more significant in comparison with the male participants (Table 5).

### 3.5. The Perception of the Significance of Cooperation in Interdisciplinary Medical Teams According to the First-Year Students

The study demonstrated that the perception of cooperation in interdisciplinary teams differs among first-year students depending on the faculty (Table 6). Additionally, first-year nursing students attributed greater significance to cooperation in interdisciplinary teams as compared to the students from other faculties.

The analysis of multiple comparisons revealed that statistically significant differences in the perception of the significance of cooperation in interdisciplinary teams were observed among first-year students of medicine and nursing (*p* < 0.001), medicine and physiotherapy (*p* = 0.001), nursing and midwifery (*p* = 0.001). No differences were found in the opinions of students from other faculties.

Statistical analysis showed that faculty did not affect the presence of significant differences in the perception of cooperation in interdisciplinary teams among the final-year students.

## 4. Discussion

We are facing unprecedented social, economic and environmental challenges. The rapid development of new technologies, globalization, population migrations, an aging society, longer life expectancy, an increase in the percentage of people suffering from chronic diseases and the deepening specialization of medical professions pose new challenges for healthcare professionals who will have to apply their knowledge and skills in unknown and changing circumstances.

Research shows that providing care by an interdisciplinary team increases the effectiveness of therapy, affects work efficiency and improves the professional relations of medical staff [29,30,31,32,33]. It seems, therefore, that an interdisciplinary approach to the patient’s health problems, in which representatives of at least two professions cooperate with each other to provide comprehensive care, could be a response to patients’ expectations and changes taking place in society.

For cooperation to be effective, medical staff should have the skill to work in a group. It is important for professionals to be able to communicate effectively with other team members, with the patient and her/his family. They should be able to define the scope of their and other professionals’ competencies and accept responsibility for participation in decision-making. Therefore, it is important to develop these skills already during undergraduate education. During university classes, students should have the opportunity to acquire knowledge, skills and social competencies necessary to undertake effective interdisciplinary cooperation. Knowing their attitudes towards interdisciplinarity will allow decision makers to better adjust teaching methods and curricula.

Recent studies on students’ attitudes towards interdisciplinary collaboration and interdisciplinary education come from the UK, USA and Canada. In order to create a curriculum, the results from those studies should be interpreted with caution: students’ educational backgrounds, as well as attitudes, expectations and stereotypes, may vary considerably between institutions and countries and may influence how interdisciplinary cooperation is defined and experienced.

In Europe, most medical university programs are public, and rather larger cohorts of students are educated (e.g., there are nine medical universities in Poland, three Collegium Medicum, five university faculties and two faculties of non-public universities where the MD program is available, and almost 4400 new medical students per educational year [34], leading to an average class size of over 230 students), while in the USA (141 fully-accredited medical schools), more than one third are private (*n*  =  56) and class size is much smaller, with an average of 146 students per educational year [35]. This may also explain the higher frequency of studies from the USA, as implementing IPE elements could be more feasible with smaller classes, and private medical schools may suffer more pressure to evaluate their programs [36].

Attention should also be paid to the scope of competencies of individual members of the therapeutic team, which most often results from the legal regulations of the country. In the traditional hierarchal model of healthcare, the physician is the primary leader and decision maker and guides the entire care journey of the patient. The physician recommends therapies, medicines and other interventions based on their clinical decision-making process. Traditionally, pharmacists, nurses, medical assistants and other therapists are left out of the decision-making process and are expected to follow through on physicians’ orders with little input. Nowadays, the traditional approach in healthcare is being rejected in favor of holistic care, in which the scope of competencies of medical staff is complementary. In the Polish healthcare system, the scope of responsibilities of a physician includes health examination, diagnosis and prevention of diseases; providing the patient or his/her statutory representative with accessible information about his/her health, diagnosis, proposed and possible diagnostic and therapeutic methods [37]. The duties of the nurse are implementation of medical recommendations in the diagnostic, treatment and rehabilitation processes; creating a nursing care plan, administration of drugs, prescribing certain medications [38]. The main duty of a midwife is to take care of a woman at every stage of her life, especially conducting a physiological delivery, diagnosing pregnancy, and caring for the patient in the course of a physiological pregnancy and postpartum [38]. The scope of responsibilities and duties of a physiotherapist includes rehabilitation of patients, conducting patient functional diagnostics, matching the patient needs and medical products, and implementation of medical recommendations in the diagnostic, treatment and rehabilitation processes [39]. Which may explain the differences in the perception of interdisciplinary cooperation among students of different faculties.

As it has been presented, some of the competencies of the therapeutic team members overlap, which creates an opportunity for effective cooperation. However, it should be noted that in the majority of Polish medical care facilities it is the physician who acts as a leader in the therapeutic team who sets the direction of medical care. Which may explain the differences in the perception of interdisciplinary cooperation by representatives of different medical faculties.

Nevertheless, it was shown that the students participating in the study positively evaluated providing patient care by the interdisciplinary team. According to their opinions, the benefits of the interdisciplinary team were rated high. Moreover, the students indicated that the therapeutic process based on the interdisciplinary model improved the quality of medical care provided, increased patient safety, improved communication between the therapeutic team members, and stimulated job satisfaction among healthcare professionals. Factors affecting the significance of cooperation in interdisciplinary medical teams also included the year of studies, the gender of the respondents, and the participation in multidisciplinary classes. The obtained results are consistent with the reports of Grace Vincent-Onabajo et al. [40]. Onabajo’s study assessed attitudes of undergraduate healthcare students in Nigeria toward interprofessional practice. A cross-sectional survey of a convenience sample of 489 dental, medical, medical laboratory science, nursing, physiotherapy and radiography students from a public university was conducted. The mean attitude score was 53.75 (± 7.41) out of 70 depicting an overall positive attitude. The year and course of study resulted in statistically significant (*p*< 0.001) differences in attitudes with students in the sixth year of study, and medical students having more positive attitudes than their colleagues in junior years and other courses, respectively. The differences were also observed in other research studies [41,42,43].

The study revealed that differences in the perception of the role played by the interdisciplinary teams and the benefits of such an approach to patient care were mainly found among nursing and medical students. Nevertheless, there are a number of factors that may contribute to such observations.

The study also indicated that gender had a statistically significant impact on the perception of providing medical care by the interdisciplinary team. In fact, women predominate in such faculties as nursing. According to the Central Statistical Office [44], in the academic year 2018/2019, 90% of nursing students were female. In contrast, in the medical faculty, female students accounted for 57% of the entire student population, which may account for the higher scores with regard to the perception of interdisciplinary teams by nursing students.

The study by Wilhelmsson et al. [45] assessing students’ readiness to cooperate in a team demonstrated that regardless of the educational program, female students evaluated teamwork higher than male students. However, in the group of female students, nursing students perceived the benefits of teamwork more positively, rated interprofessional education higher and showed a greater readiness to cooperate with the representatives of other faculties in comparison with the medical students.

The differences in opinions may also stem from the social perception of the two professions, as well as from the tradition associated with the history of their development. Until recently, nurses were perceived as physician’s assistants. Nowadays, they have become independent medical professionals, legitimate members of the therapeutic team and fully responsible for the medical care they provide. Such an image of contemporary nurses has been promoted among practicing nurses, as well as among persons preparing to practice this profession. However, as indicated in the study by Malik et al. [46], the image of a nurse as a subordinate to a physician is still present both among the representatives of other medical professions and in society. Medical students participating in the study by Malik et al. indicated that the majority of physicians did not perceive the essential role played by a nurse in patient care, thus, diminishing the nurse’s status in the therapeutic team. According to the respondents, the cooperation between a physician and a nurse is frequently inadequate and results from the lack of openness, mutual resentment and deeply rooted stereotypes.

Another reason accounting for different perceptions of interdisciplinary teams by nursing and medical students may be due to a different professional culture, as pointed out by Hall. According to her, each medical profession has its own culture, which includes beliefs, customs and behaviors. Therefore, the traditional approach to the organization of the educational process, in which students participate in classes only with students of their year, reinforces the shared views of a given group, the sense of belonging to the specific, individual professional community, as well as the development of a specific approach to problem solving. Additionally, it also shapes the distinctive language/jargon of each professional group [47]. According to Hall, in the education of medical students, the responsibility for the decision-making process is highly emphasized, which is also associated with assuming a leadership role. Hence, it may be challenging for such students to acquire the skills required to work in an interdisciplinary team, where leadership is based on the collaboration of several individuals. Moreover, failure to recognize the values shared by the professional group may also result in conflicts and communication difficulties.

Furthermore, differences in emotional and social competencies also constitute a factor contributing to a different perception of the role of interdisciplinary teams and their significance among medical and nursing students. The study conducted by Tyszkiewicz-Bandur et al. [48] showed that medical students obtained poorer scores with respect to all emotional intelligence domains as compared to students of the Faculty of Health Sciences (including nursing, midwifery, health promotion studies, cosmetology, emergency medicine and dietetics). Additionally, the scores of medical students were also lower in terms of social competence in intimate situations. In fact, social competence scores in intimate situations have been fundamental to the helping professions within which the medical profession operates, since they refer to the skills involving the establishment of emotionally deep relationships. Moreover, medical students also obtained poorer scores with respect to emotional intelligence, both in the interpersonal and intrapersonal areas. Therefore, they may experience difficulties recognizing the emotional states not only of others but also of their own. In fact, the results may suggest that medical students tend to present a higher degree of individualism than students from the other faculties involved in the study.

Therefore, to ensure that students acquire the skills indispensable for teamwork, it is vital to create a curriculum where they can learn both with and about one another, solve problems together, as well as exchange experiences and knowledge. These assumptions are fulfilled by interprofessional education.

Interprofessional education of medical students prior to obtaining their license to practice positively affects their perception of students from other faculties, improves communication, and provides greater training to function as a member of the interdisciplinary healthcare team [49]. The above-mentioned conclusions were also confirmed in the present study. It was demonstrated that students participating in classes with students from other medical faculties and during which teachers employed methods encouraging interaction between students of different faculties, rated higher the advantages of cooperation in interdisciplinary medical teams.

Several authors have suggested that in order to eliminate stereotypes, which may negatively affect the readiness of students to cooperate with representatives of other medical faculties, education based on the interdisciplinary model should start as early as the first years of studies [50,51,52]. In fact, the study by Rudland and Mires [53] indicated that stereotypes among medical students stemmed from social perceptions of medical professions rather than being acquired in the course of education. Therefore, interprofessional education introduced early in academic training will not affect the formation of stereotypes.

According to Gilbert [50], it is most effective to introduce interprofessional education elements into the curricula in the course of the first years of study, as well as to establish the curricula in the final year according to the principles of interprofessional education. Primarily, it allows the students to develop their professional identity and identification with their own profession. Additionally, it also shapes the ability to work in a team and develops an attitude of openness towards representatives of other medical professions.

In contrast, research indicates that introducing interprofessional education too late may result in the reluctance of students to interact with others due to the development of a profession-specific approach to solving clinical problems and the reinforcement of stereotypes and prejudices [54]. Thus, although the most effective time to introduce interdisciplinary elements into medical education has not been determined, the solution proposed by Gilbert appears to be the most favorable.

Nevertheless, developing curricula for medical students which would incorporate interprofessional education may prove to be a major challenge. The complexity of the simultaneous teaching of students from different faculties, logistic and organizational issues or overcrowded timetables may constitute factors hindering the implementation of interprofessional education. However, providing students with the knowledge, skills and social competence essential for professional and holistic care and addressing current health issues within society, constitute the primary objectives and should be accomplished at the level of academic education.

A factor supporting the introduction of interprofessional education, which shapes the ability to work in an interdisciplinary team, is the fact that medical students perceive the significance of interdisciplinary collaboration and recognize its benefits—both for patients and for medical professionals, as demonstrated in this study. Nevertheless, in order to be effective, the curricula should also account for the differences in the perception of cooperation in interdisciplinary teams. In this aspect, it is essential to respect the diversity of students depending on their faculty, as well as to account for the impact of gender and the year of studies on their readiness to engage in team collaboration. Teachers conducting multidisciplinary classes should also be aware of the existing differences, stereotypes and prejudices among the students. Using the available didactic methods, the lecturers should deliberately engage the students in successful cooperation which will contribute to the improvement in the quality of patient care in the future.

## 5. Conclusions

The students participating in the study perceived the interdisciplinary cooperation in healthcare teams as significant. The differentiating factors in their perception of collaboration in interdisciplinary healthcare teams included the faculty and the year of studies, gender, and participation in multidisciplinary courses.

The students who attended multidisciplinary classes were more likely to perceive the benefits of medical care provided by the interdisciplinary team in comparison to the students who did not participate in such activities. Therefore, the introduction of the academic courses based on the principles of interprofessional education may contribute to providing students with the skills essential to engage in effective teamwork.

## Figures and Tables

**Table 1:** The characteristics of the studied group.

Characteristics	*n* (%)
Gender	
Woman	1036 (81.8%)
Man	226 (17.9%)
Other	4 (0.3%)
Age	
18–20 years	724 (57%)
21–23 years	353 (28%)
24 and older	189 (15%)
Year and field of study	
1st-year medicine students	205 (16%)
6th-year medicine students	103 (8%)
1st-year midwifery students	247 (20%)
3rd-year midwifery students	101 (8%)
1st-year nursing students	162 (13%)
3rd-year nursing students	154 (12%)
1st-year physiotherapy students	220 (17%)
3rd-year physiotherapy students	74 (6%)
Place of residence	
A village	364 (29%)
A city below 50,000 inhabitants	296 (23%)
A city between 50,000 and 500,000 inhabitants	271 (21%)
A city of more than 500,000 inhabitants	335 (27%)
University classes in interdisciplinary student groups	
Standard curriculum classes	468 (37%)
Elective courses	430 (34%)
Professional training	316 (25%)
Workshops at a conference	164 (13%)
PUMS projects	152 (12%)
Other	38 (3%)

**Table 2:** The impact of the faculty on the perception of the significance of collaboration in interdisciplinary medical teams.

Faculty	*n*	Mean ± SD	*p*-Value
Medicine	308	3.89 ± 0.47	<0.001
Nursing	316	4.03 ± 0.39	
Midwifery	348	3.90 ± 0.37	
Physiotherapy	294	3.97 ± 0.41	
Total	1266	3.95 ± 0.41	

**Table 3:** The impact of the year of studies on the perception of the significance of cooperation in interdisciplinary medical teams.

Year of Study	*n*	Mean ± SD	*p*-Value
1st year	834	3.83 ± 0.38	<0.001
Final year	432	4.16 ± 0.40	

**Table 4:** The perception of the significance of cooperation in interdisciplinary medical teams according to the participation in multidisciplinary courses.

The Participation in Multidisciplinary Courses	*n*	Mean ± SD	*p*-Value
Yes	431	4.00 ± 0.41	0.004
No	835	3.92 ± 0.41	

**Table 5:** The perception of the significance of cooperation in interdisciplinary medical teams according to gender.

Gender	*n*	Mean ± SD	*p*-Value
woman	1036	3.96 ± 0.40	0.002
man	226	3.86 ± 0.46	

**Table 6:** The perception of the significance of cooperation in interdisciplinary healthcare teams among first-year students according to the faculty.

Faculty	*n*	Mean ± SD	*p*-Value
Medicine	205	3.74 ± 0.43	<0.001
Nursing	162	3.95 ± 0.33	
Midwifery	247	3.79 ± 0.32	
Physiotherapy	220	3.88 ± 0.38	
Total	834	3.83 ± 0.38	

## Data Availability

The data presented in this study are available in Supplementary Material.

## Supplementary Materials

The following are available online at https://www.mdpi.com/article/10.3390/ijerph20020954/s1.
